# Oncogenic epigenetic control

**DOI:** 10.18632/aging.100940

**Published:** 2016-04-01

**Authors:** Gary S. Stein, Terri L. Messier, Jonathan A. R. Gordon, Joseph R. Boyd, Coralee E. Tye, Jane B. Lian, Janet L. Stein

**Affiliations:** Department of Biochemistry and University of Vermont Cancer Center, University of Vermont College of Medicine, Burlington, VT, 05405, USA

**Keywords:** epigenetic control, chromatin, breast cancer

There is growing recognition that epigenetic regulation contributes significantly to the fidelity of biological control. Compromised epigenetic pathways have now been shown to provide indications of transformation and tumor progression; they suggest potential targets for cancer prevention and intervention [1]. A formidable challenge is to deconvolute the complexity and plasticity of histone modifications associated with tumorigenesis. Although challenging, identification of epigenetic signals that are functionally associated with oncogenesis will provide a better understanding of the mechanisms underlying their impact.

Post translational modifications of histone3 lysine4 (H3K4) may constitute epigenetically mediated regulatory signatures that can be informative for the molecular characterization of breast cancer pathology. Both methylation and acetylation of H3K4 are associated with gene activation. Tri-methylation of histone3 lysine4 has been extensively studied and is a well-documented marker for active or poised gene transcription. The enzyme complexes that regulate H3K4me3 have been implicated in tumorigenesis, pointing to the relevance of this epigenetic modification in cancer. Histone acetylation as well as histone acetyltransferases and deacetylating enzymes are recognized as important in tumor progression. However, the acetylation of H3K4 in particular has been less extensively investigated to date.

Messier, et al., addressed the coordinated contributions of H3K4me3 and H3K4ac to breast cancer tumorigenesis in three well-established human mammary cell lines that represent a normal-like subtype (MCF10A; fibrocystic disease) and two cancer subtypes, luminal (MCF7; ER+/PR+) and basal-like metastatic (MDA-MB-231; ER−/PR−/HER2−) [2]. These cell lines recapitulate the phenotypic and proliferative transitions that accompany tumor progression states. Utilizing ChIP-seq analysis, a genome-wide map of H3K4me3 and H3K4ac patterns was developed, establishing dynamic alterations in the overall histone H3K4 epigenetic landscape in the three cell lines. An observed global increase in H3K4ac in MCF7 and MDA-MB-231 cell lines that is accompanied by a global increase in H3K4me3 in the MDA-MB-231 metastatic cell line is consistent with increased representation of both histone marks during oncogenic progression. Modifications in H3K4 tri-methylation and acetylation were found to occur on specific chromosomes, including the X-chromosome. This striking change in H3K4 tri-methylation and acetylation may account for the loss of X-chromosome inactivation and epigenomic instability of the inactive X-chromosome seen in breast cancer [3].

In contrast to methylation and acetylation of H3K9 and H3K27, which activate or repress transcription respectively, methylation and acetylation of H3K4 are both associated with gene activation. Observed increases in H3K4 acetylation marks at specific gene promoters in both the MCF7 and MDA-MB-231 cell lines, compared to normal-like MCF10A cells, suggests that H3K4 acetylation may be an early step in cancer initiation or progression. The global increase in H3K4me3 at gene promoters in MDA-MB-231 cells suggests alterations in the epigenetic landscape that may be functionally related to late stage, metastatic breast cancer.

From a mechanistic perspective, the presence of H3K4ac and H3K4me3 at specific gene promoters provides a blueprint for regulatory pathways that are functionally linked to epigenetic control of breast cancer onset and progression. The dynamic acetylation of H3K4 is associated with estrogen-receptor responsiveness in the MCF7 cells and the initial loss of mammary tissue–specific gene expression in MCF7 cells that represent early stage breast cancer. Enhancing the pivotal role of H3K4ac in initial stages of breast cancer tumorigenesis, this epigenetic mark appears to poise genes for expression that leverages continued acquisition and persistence of metastatic properties. In contrast, H3K4me3, with persistence of H3K4ac reflects the epithelial to mesenchymal transition in MDA-MB-231 cells that represent late stage breast cancer. Important mechanistic linkages between histone H3K4ac and H3K4me3 modifications and breast cancer tumorigenesis remain to be established.

While H3K4ac is emerging as an informative predictor of pathways that are deregulated during the onset and progression of breast cancer, the full potential for exploitation of this epigenetic modification as a biomarker and therapeutic target remains to be explored. Future studies must further define the enzymes that support acetylation and deacetylation of H3K4. Furthermore, the dynamic balance between H3K4 acetylation and methylation must be elucidated within the context of the full spectrum of histone modifications that contribute to combinatorial control of the epigenetic landscape. This will provide a drastically improved understanding of how epigenetic modifications and chromatin organization regulate mammary epithelial phenotype and gene expression related to transformation and tumor progression—the complexity of chromatin organization that supports breast cancer initiation and progression is increasingly evident. Consequently, the clinical value of epigenetic biomarkers and related therapeutic targets can be expanded by detailed characterization of the regulatory signals responsible for the deposition of specific “histone marks”. Adding to the biological challenge inherent in these analyses, the plasticity observed in post translational histone modifications must also be considered. It will be important to establish the relationships between histone modifications and other parameters of epigenetic control and chromatin organization in breast cancer that include perturbations in inter- and intra-chromosomal interactions [4] and oncofetal recapitulation of bivalent epigenetic regulatory mechanisms [5-8]. Although challenging, the complexity of epigenetic control in breast cancer provides the potential for increased capabilities in breast cancer risk assessment, early diagnosis and therapy with enhanced specificity and minimal off-target consequences.

**Figure 1:**
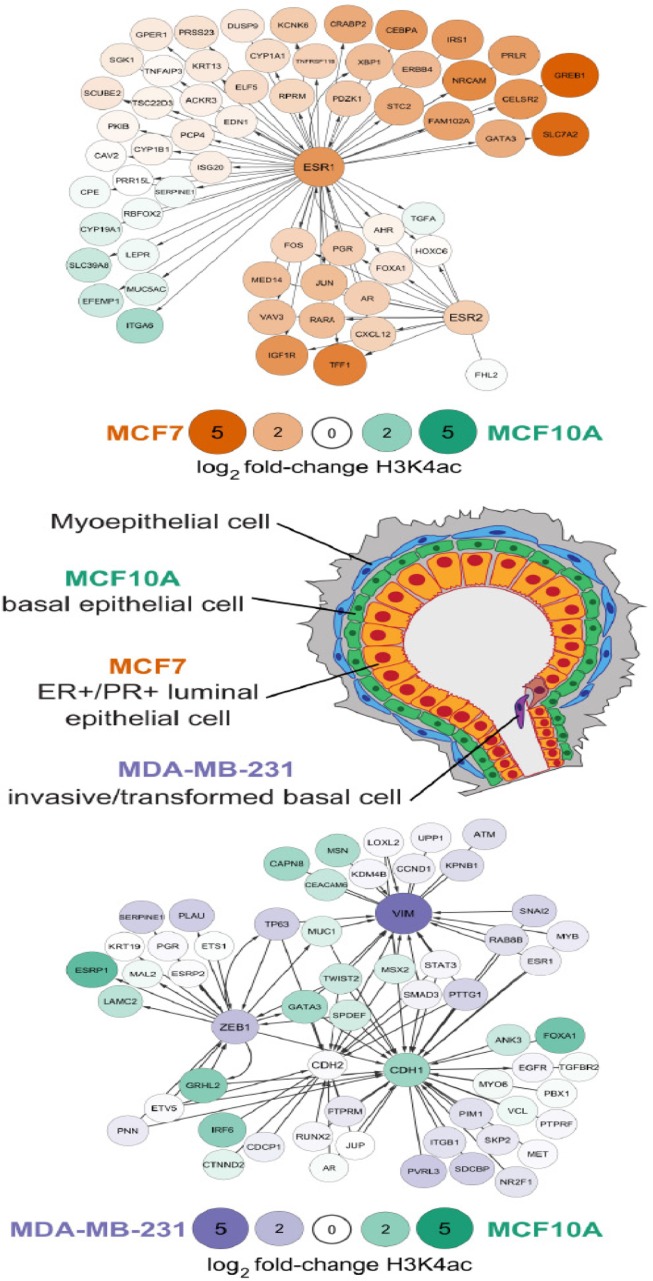
Dynamics of H3K4ac epigenetic modifications in cell lines representative of breast/breast cancer subtypes The central image represents the epithelial layers in a human breast duct and the cell lines used in the study discussed here. MCF10A is a normal-like basal epithelial–derived cell line; MCF7 represents the ER+/PR+ luminal-cell subtype, correlative with a relatively favorable prognosis; and MDA-MB-231 is representative of the ER−/PR−/Her2− metastatic basal cell subtype, correlative with a less favorable prognosis. The upper network shows changes in H3K4ac between MCF10A and MCF7 for genes involved in estrogen-response. The lower network compares H3K4ac between MCF10A and MDA-MB-231 for genes involved in the epithelial to mesenchymal transition. Differences in enrichment between cell lines as indicated by color and size of nodes.
